# miR-379-5p promotes ovarian granulosa cell apoptosis in primary ovarian insufficiency by targeting KNDC1 and PEG10

**DOI:** 10.3389/fgene.2026.1827032

**Published:** 2026-05-13

**Authors:** Jie Luo, Fan Zhang, Yurong Feng, Fang You, Daobin Yang, Zengguang Wu, Li Zeng

**Affiliations:** 1 Guizhou University of Traditional Chinese Medicine, Guiyang, China; 2 Second Clinical Medical College of Guizhou University of Traditional Chinese Medicine, Guiyang, China; 3 Reproductive Medicine Department, Guizhou Provincial People’s Hospital, Guiyang, China

**Keywords:** apoptosis, KNDC1, miR-379-5p, ovarian granulosa cells, PEG10, primary ovarian insufficiency

## Abstract

**Background:**

Primary ovarian insufficiency (POI) is a heterogeneous disorder characterized by premature decline in ovarian function in women of reproductive age, yet its molecular mechanisms remain incompletely elucidated. MicroRNAs (miRNAs), as crucial post-transcriptional regulators, may play a crucial role in the onset and progression of POI. Our research sought to elucidate the regulatory roles and mechanisms of key miRNAs and their target genes in ovarian granulosa cells (GCs) from POI patients.

**Methods:**

Based on POI-related mRNA and miRNA microarray datasets obtained from GEO database, we employed bioinformatics methods to identify differentially expressed genes (DEGs) and performed functional enrichment analyses. The interactions between miRNAs and target genes were validated using dual-luciferase reporter assays. In POI cell models, following transfection with miRNA mimics or inhibitors using a lipid-based reagent, we assessed their effects on cell proliferation, apoptosis, and cell cycle progression using CCK-8 assays, flow cytometry, real-time quantitative PCR (qRT-PCR), and Western blotting. Finally, rescue experiments were conducted to further validate the underlying mechanism.

**Results:**

This study identified 590 DEGs and 610 differentially expressed miRNAs (DEMs) from microarray datasets, which were primarily enriched in processes including cell cycle regulation, chromosome segregation, and tubulin binding. Notably, miR-379-5p was significantly upregulated in the POI group. Intersection analysis of DEGs and predicted miR-379-5p targets identified two key genes, Kinase Non-catalytic C-lobe Domain Containing 1 (KNDC1) and Paternally Expressed Gene 10 (PEG10). Experimental validation confirmed that miR-379-5p was highly expressed in the POI GCs and directly targeted and suppressed the expression of KNDC1 and PEG10. miR-379-5p mimics reduced cell viability, increased apoptosis, and induced G0/G1 phase arrest. Conversely, miR-379-5p inhibition or KNDC1/PEG10 overexpression reversed these phenotypic changes.

**Conclusion:**

This study revealed elevated miR-379-5p expression in POI GCs, which induced cell cycle arrest and apoptosis by suppressing the expression of KNDC1 and PEG10. These findings provide a theoretical basis for understanding POI pathogenesis and for developing targeted therapeutic strategies centered on miR-379-5p.

## 1 Introduction

Primary ovarian insufficiency (POI) is an endocrine disorder, manifesting as a premature decline of ovarian functioning among women of reproductive age. Its clinical features include amenorrhea, infertility, elevated gonadotropin levels, and decreased estrogen levels (Rahman and Panay, 2021). The impact of POI extends beyond fertility issues, as it also significantly increases the risk of osteoporosis and cardiovascular disease (Huang et al., 2022). The etiology of POI is complex and involves genetic factors (e.g., X chromosome abnormalities, single-gene mutations) (Akande et al., 2020), autoimmune disorders (Silva et al., 2014), iatrogenic damage (e.g., chemotherapy, radiation, or ovarian surgery) (Rossetti et al., 2017), environmental toxin exposure, and metabolic dysfunction. Currently, the predominant treatment for POI is hormone replacement therapy (HRT) (Sarrel et al., 2016). However, HRT is effective in alleviating symptoms and improving quality of life; it cannot restore fundamental ovarian functions including hormone secretion, follicular growth, or ovulation (Takahashi et al., 2021). Consequently, elucidating the pathological mechanisms behind POI is crucial for devising effective targeted therapeutic strategies.

MicroRNAs (miRNAs) are a class of small non-coding RNA molecules, typically 18–25 nucleotides in length, and play a critical role in regulating gene expression. They negatively regulate gene expression by binding to the 3′untranslated region (3′UTR) of target mRNAs, thereby inhibiting protein translation or promoting mRNA degradation (Agbu and Carthew, 2021). Recent studies on female reproduction have indicated that miRNAs are key regulators of ovarian physiological functions (Pankiewicz et al., 2021). They contribute to the maintenance of normal ovarian activity by modulating ovarian granulosa cell (GC) performance (Sun et al., 2019), follicular development, steroid hormone synthesis, and oocyte maturation (McGinnis et al., 2015). For instance, miRNA-22-3p affects FSH signaling and downregulates the expression of ESR1/PTEN, thereby inducing GC apoptosis (Qin et al., 2015). Furthermore, miR-146a exerts pro-apoptotic effects through targeting TRAF6 and IRAK1 (Chen et al., 2015). miR-383 and miR-320 regulate ovarian steroidogenesis by targeting the estrogen receptor-related transcription factor E2F1 (Yin et al., 2014). Individual miRNA simultaneously targets multiple mRNAs, while multiple miRNAs can work together to regulate a single gene, leading to a complex “one-to-many” and “many-to-one” relationship that constitutes an intricate gene regulatory network (Sheikhansari et al., 2018). From a systems biology perspective, our current understanding of the POI-related miRNA-mRNA regulatory network remains incomplete and lacks systematic characterization, leaving key regulators and their underlying mechanisms largely unexplored.

High-throughput sequencing and bioinformatics analyses represent powerful tools for systematically exploring molecular mechanisms underlying diseases (Capar Gorali et al., 2023). To investigate the POI-related miRNA-mRNA interaction network, we integrated mRNA and miRNA microarray datasets related to POI from the GEO database, enabling to construct a disease-specific miRNA-mRNA regulatory network. Focusing on the significantly upregulated miR-379-5p, we validated the regulator its roles in regulating target genes, KNDC1 and PEG10, which involved in apoptosis and cell cycle progression in POI GCs. The present work seeks to offer new theoretical perspectives on the pathogenesis of POI and to lay a foundation for future therapies targeting miR-379-5p.

## 2 Materials and methods

### 2.1 Analysis of POI-related mRNA and miRNA microarray datasets

Microarray datasets for POI-related mRNA (GSE201276) and miRNA (GSE100238) were derived from GCs of POI patients and healthy controls, respectively, and were downloaded from the GEO database. The GEO2R online tool was used for the initial identification of DEGs, with thresholds set at |logFC| > 1 and P < 0.05. To enhance the reliability of screening results, differential expression analysis was performed concurrently using three algorithms: edgeR, limma, and DESeq2, and the intersection of their outputs was taken as the final set of DEGs and DEMs. All subsequent analyses were based on these DEGs and DEMs screened in the previous procedures. Volcano plots were generated using R, and functional enrichment analyses including Gene Ontology (GO) classification, KEGG pathway enrichment, and Reactome pathway analysis were performed through Metascape platform. Finally, a protein-protein interaction (PPI) network was constructed for the top 20 most significantly DEGs using the STRING database.

DEMs were identified following the same methodology previously described. The multiMiR package (v1.20.0, including 14 databases such as DIANA-microT-CDS, ElMMo, miRTarBase, miRanda, miRDB, PicTar, and TargetScan.) was employed to predict the target mRNAs for these DEMs. A miRNA-mRNA regulatory network was constructed using Cytoscape. Additionally, a Venn diagram was generated through the VENNY 2.1 online tool to identify overlaps between DEMs and their predicted target mRNAs, facilitating the screening of core regulatory pairs associated with POI.

### 2.2 Cell lines and culture conditions

Human GC line KGN (Beina Bio, BNCC337610) was cultured in DMEM/F12 complete medium (GIBCO, 11330032) supplemented with 10% fetal bovine serum (Sigma, 12003C), and maintained in a humidified incubator (Thermo Scientific, 311) at 37 °C with 5% CO_2_. For subsequent experiments, KGN cells at logarithmic growth phase were treated with 4-vinylcyclohexene diepoxide (VCD) (Aladdin, V102412) at a final concentration of 2 mM for 24 h, followed by analyses. KGN cells treated with VCD were used as the POI-related GC model (MOD), while untreated KGN cells served as the negative control group (NC) (Zhou et al., 2023).

### 2.3 RNA extraction and quantitative real-time RT-PCR

GCs were harvested and total RNA was isolated using TRIzol® reagent (CoWin Biosciences, CW0580S) according to the manufacturer’s instructions. The concentration and purity of the RNA were measured using a NanoDrop One/One microspectrophotometer (Thermo Scientific). Reverse transcription was performed using the Goldenstar™ RT6 cDNA Synthesis Kit Ver.2 (Tsingke, TSK302M). The reaction mixture was prepared following the kit protocol, and the reverse transcription program was set as follows: 25 °C for 10 min, 55 °C for 15 min, 85 °C for 5 min, followed by holding at 4 °C. The resulting cDNA was used as the template for qPCR. The qPCR reaction mixture (20 μL) was prepared using the 2 × T5 Fast qPCR Mix (SYBR Green I) kit (Tsingke, TSE202) and contained 10 μL of 2 × T5 Fast qPCR Mix, 0.5 μL each of forward and reverse primers (10 μM), 1.0 μL of cDNA template, and ddH_2_O to bring the final volume to 20.0 μL. The qPCR was carried out on a Bio-Rad IQ5 real-time PCR system using a two-step amplification protocol: initial denaturation at 95 °C for 30 s, followed by 40 cycles of 95 °C for 5 s and 60 °C for 30 s. GAPDH was used as the reference gene for mRNA, and U6 was used as the reference gene for miRNA. The relative expression levels of target genes were calculated using the 2^–ΔΔCt^ method. The primer sequences are listed in Table 1. The amplification curves, melting curves, and agarose gel electrophoresis results of the PCR products are shown in the Supplementary Material S1.

**TABLE 1:** Primer sequences.

Terms	Sequence
miR-379-5p	F:GTGGTAGACTATGGAACGTAGG R:TACGTTCCATAGTCTACCA
U6	F:CTCGCTTCGGCAGCACA R:ACGCTTCACGAATTTGCGT
KNDC1	F:GTGACCGGGAACACCTTTGA R:AATGGCAGAGGCCACCATAG
PEG10	F:CTCGCGTGAAATAAGCGGGT R:TTCGGTCATGTTGGGGACAC
CYP19A1	F: ATGAAAGCTCTGTCAGGCCC R: TCAACACGTCCACATAGCCC
FSHR	F: GCCAAGAGAGCAAGGTGACA R: ACTCGAAGCTTGGTGAGGAC
StAR	F: CAGACTTCGGGAACATGCCT R: GGGACAGGACCTGGTTGATG
GAPDH	F:TCAAGGCTGAGAACGGGAAG R:TCGCCCCACTTGATTTTGGA

### 2.4 Western blot

Total protein was extracted using RIPA buffer (Beyotime, P0013B) supplemented with PMSF and a protease inhibitor cocktail (Beyotime, P1045). Protein concentration was determined using the BCA method (Thermo Scientific, A55861). An appropriate amount of protein was resolved by SDS-PAGE to separate the proteins based on their molecular weight. After electro-transfer of proteins onto 0.22 µm PVDF membranes, the membranes were blocked with 5% skim milk (dissolved in TBST containing 0.1% Tween-20) for 1 h at room temperature. Subsequently, the samples were incubated with primary antibodies overnight at 4 °C, followed by incubation for 2 h at room temperature with horseradish peroxidase-conjugated secondary antibody. Signals were developed with an ECL substrate (Thermo Scientific, 34580) and captured using a gel imaging system (Bio-Rad, Universal Hood II). All antibodies were diluted in antibody diluent (Abclonal, RM02955) at the indicated ratios. The primary antibodies used were: PEG10 (1:1000, Immunoway, YM1073), KNDC1 (1:500, Bioss, bs-10056R) and GAPDH (1:5000, Abclonal, A19056). The secondary antibodies were HRP-conjugated goat anti-rabbit IgG (1:5000, Abclonal, AS014) and HRP-conjugated goat anti-mouse IgG (1:5000, Abclonal, AS003).

### 2.5 miRNA mimics/inhibitor synthesis and transfection

miR-379-5p mimics, mimics negative control (mimics-NC), miR-379-5p inhibitor, and inhibitor negative control (inhibitor-NC) were synthesized by Chongqing Biomedicine Biosciences Co., Ltd. Transfection was performed using the lipid-based transfection reagent Max (Thermo Scientific, 16447100). Each of the mimics-NC, miR-379-5p mimics, inhibitor-NC, and miR-379-5p inhibitor was prepared by dissolving in an appropriate volume of Opti-MEM medium (Gibco, 31985070) and incubating at room temperature for 5 min. Subsequently, the diluted solution was mixed with Opti-MEM containing Max transfection reagent and allowed to stand at room temperature for 5 min to form transfection complexes. The complexes were evenly added to the cell culture dishes. Six hours post-transfection, the medium was replaced with fresh complete medium. After an additional 48 h of incubation, cells were harvested for subsequent assays. The sequences of miR-379-5p mimics and inhibitor are listed in Table 2.

**TABLE 2:** miRNAs-mimics/inhibitor Sequences.

Name	Sequence
Mimics NC	F: UCACAACCUCCUAGAAAGAGUAGA R: UCU​ACU​CUU​UCU​AGG​AGG​UUG​UGA
miR-379-5p mimics	F: UGGUAGACUAUGGAACGUAGG R: CCU​ACG​UUC​CAU​AGU​CUA​CCA
NC-inhibitor	UCUACUCUUUCUAGGAGGUUGUGA
miR-379-5p inhibitor	CCUACGUUCCAUAGUCUACCA

### 2.6 Dual-luciferase reporter assay

Potential binding sites for miR-379-5p within the 3′UTR of KNDC1 and PEG10 were predicted using TargetScanHuman 8.0 database. DNA fragments containing wild-type (WT) or mutant (Mut) sequences of the putative miR-379-5p binding sites in the KNDC1 and PEG10 3′UTRs were cloned into the pmirGLO dual-luciferase reporter vector, which generated the following constructs: pmirGLO-3′UTR-KNDC1-WT, pmirGLO-3′UTR-KNDC1-Mut, pmirGLO-3′UTR-PEG10-WT, and pmirGLO-3′UTR-PEG10-Mut. Mutant sequences were designed to disrupt base pairing with the miRNA seed region: the binding site sequence “TCTACCA” in KNDC1 was mutated to “CACGAAG” while “GTCTACC” in PEG10 was mutated to “ACACGAA”. (The complete sequences of the WT and Mut 3′UTR inserts are provided in Supplementary Material S2). HEK-293T cells were co-transfected with the constructed reporter vectors and either miR-379-5p mimics or negative control mimics (mimics-NC) using Max transfection reagent (Biomedicine, 24765-1). After 48 h of transfection, luciferase activity was measured using a Dual-Luciferase Reporter Assay Kit (Beyotime, RG027) according to the manufacturer’s instructions.

### 2.7 Overexpression plasmid construction and transfection

Overexpression plasmids targeting KNDC1 and PEG10 were constructed by General Biol (Anhui, China). After quality verification, they were transformed into Stbl3 competent cells (TsingKe, DLC106) using the calcium chloride method. Positive clones were selected from agar plates containing selective antibiotics. Single colonies were picked and expanded in LB liquid medium, and plasmids were extracted using a plasmid extraction kit (CWBio, CW2581S). Transfection of the overexpression plasmids was performed using a lipid-based transfection reagent Max (Thermo Scientific, 16447100). The empty vector control (oe-NC), KNDC1 overexpression plasmid (oe-KNDC1), and PEG10 overexpression plasmid (oe-PEG10), were each diluted in Opti-MEM medium (Gibco, 31985070), mixed gently and incubated at room temperature for 5 min. Each dilution was then combined with Opti-MEM containing the Max transfection reagent, gently blended, and allowed to stand at room temperature for 5 min to form transfection complexes. The complexes were uniformly added to the cell culture dishes. Fresh medium was introduced 6 h after transfection, and samples were collected 48 h later for subsequent analyses.

### 2.8 Cell viability determination by CCK-8

Logarithmic-growth phase cells were plated into 96-well plates at 5 × 10^3^ cells per well. After 24 h of culture, 10 μL of CCK-8 reagent (Beyotime, C0038) was added to each well, followed by incubation for 1 h. Absorbance at 450 nm was quantified using a microplate reader (Molecular Devices, CMax Plus).

### 2.9 Cell cycle profiling by flow cytometry

Cell suspensions were harvested and centrifuged at 2000 rpm for 5 min to obtain pellets. Following washed twice with ice-cold PBS, with supernatant discarded after each wash, fixation was performed using 1 mL of ice-cold 70% ethanol with gentle vortexing, then maintained at 4 °C for 2 h. After fixation, the cells were washed twice with pre-cooled PBS. The supernatant was discarded, and 0.5 mL of Propidium Iodide (PI) Staining Solution (Liji Bio, AC12L543) was added to each sample. Staining was performed at 37 °C protected from light for 30 min. Cell cycle profiles were acquired by flow cytometer (Changzhou BeamCyte), and data were processed with FlowJo software.

### 2.10 Flow cytometry for apoptosis detection

Cells were collected, then washed twice with ice-cold PBS, and resuspended in 100 µL of 1× binding buffer. Subsequently, 5 μL of Annexin V-FITC and 10 μL of Propidium Iodide (PI) Staining Solution (Yeasen Biotechnology, 40302ES50) were added to each sample and mixed gently. Samples were incubated 15 min at room temperature while being protected from light. Then, each tube received 400 μL of 1× Binding Buffer, followed by gentle agitation and immediate placement on ice. Apoptotic populations were quantified by flow cytometry (Changzhou BeamCyte), with data analyzed using FlowJo software.

### 2.11 Statistical analysis

All statistical analyses were performed using GraphPad Prism software, version 9.5. Data are presented as mean ± standard deviation from three independent experiments (n = 3). Statistical analysis was performed using unpaired Student's t tests for comparing two selected groups. For comparisons involving more than two groups, one-way ANOVA methods were employed, followed by Tukey’s multiple comparison tests. A p-value of less than 0.05 was considered statistically significant.

## 3 Results

### 3.1 Differential expression and functional enrichment analysis of mRNAs

**FIGURE 1:**
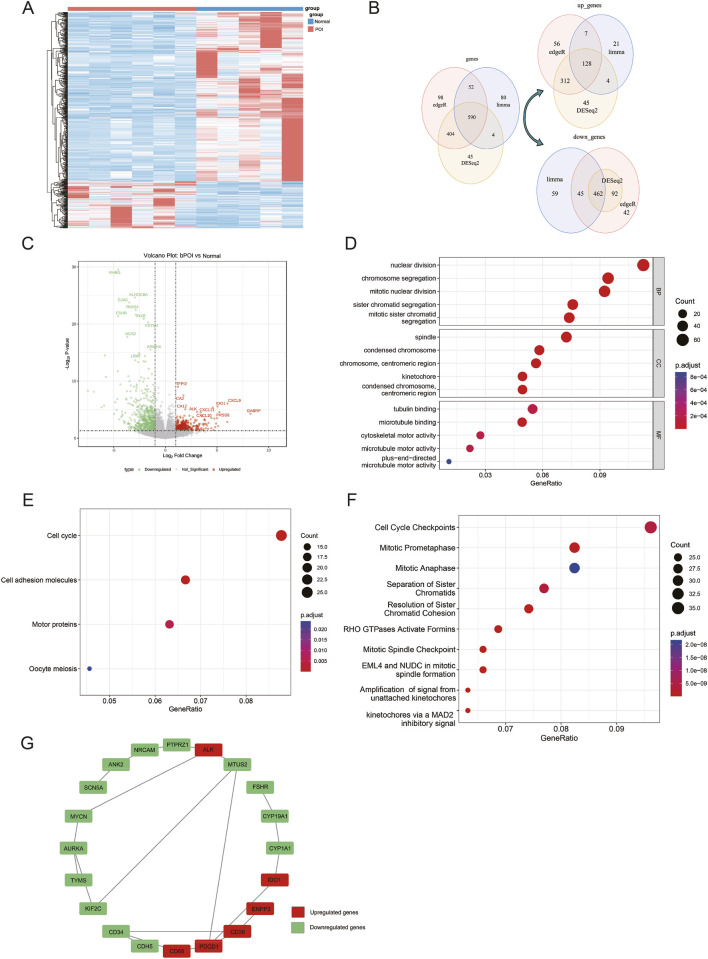
Screening and functional enrichment analysis of DEGs in the POI-related mRNA dataset. **(A)** mRNA expression heatmap comparing normal and POI groups; **(B)** Venn diagram showing common DEGs identified by edgeR, limma, and DESeq2 algorithms; **(C)** Volcano plot of DEGs; **(D)** GO enrichment analysis of DEGs; **(E)** KEGG pathway enrichment analysis of DEGs; **(F)** Reactome pathway enrichment analysis of DEGs; **(G)** PPI network of the top 20 DEGs.

To investigate the molecular characteristics of POI at the transcriptome level, we obtained mRNA datasets from GCs of POI patients and healthy controls that are accessible in the GEO database. The heatmap of DEGs revealed a distinct separation between the two groups, indicating that POI significantly impacted gene expression in affected individuals (Figure 1A). To enhance the reliability of the screening results, we employed three differential analysis algorithms: edgeR, limma, and DESeq2. This comprehensive approach identified 1144, 726, and 1043 DEGs, respectively. By intersecting these results, we obtained a total of 590 common DEGs, including 128 upregulated genes and 462 downregulated genes (Figure 1B). Volcano plot displayed the top 10 upregulated and downregulated differentially expressed mRNAs (Figure 1C).

Functional enrichment analyses (including GO, KEGG, and Reactome) were performed to investigate the potential biological functions of the DEGs. GO analysis indicated that the DEGs were mainly enriched in processes including nuclear division, spindle, and tubulin binding (Figure 1D). Meanwhile, KEGG pathway analysis indicated that DEGs were primarily associated with signaling pathways related to the cell cycle, cell adhesion molecules, and motor proteins (Figure 1E). Reactome analysis further revealed enrichment of these genes in biological processes including cell cycle checkpoints and mitotic prometaphase (Figure 1F). Furthermore, we constructed a PPI network based on the top 20 differentially expressed mRNAs, revealing two notable subgroups: one involved in cell cycle regulation (comprising MYCN, AURKA, TYMS, and KIF2C) and another related to hormone synthesis (including FSHR, CYP19A1, and CYP1A1). These genes showed interactions within the network (Figure 1G), suggesting that they might function synergistically in the pathogenesis of POI. The above results indicate that POI is closely associated with cell cycle disruption and hormone synthesis abnormalities at the mRNA level.

### 3.2 Target genes of DEMs and functional enrichment analysis

**FIGURE 2:**
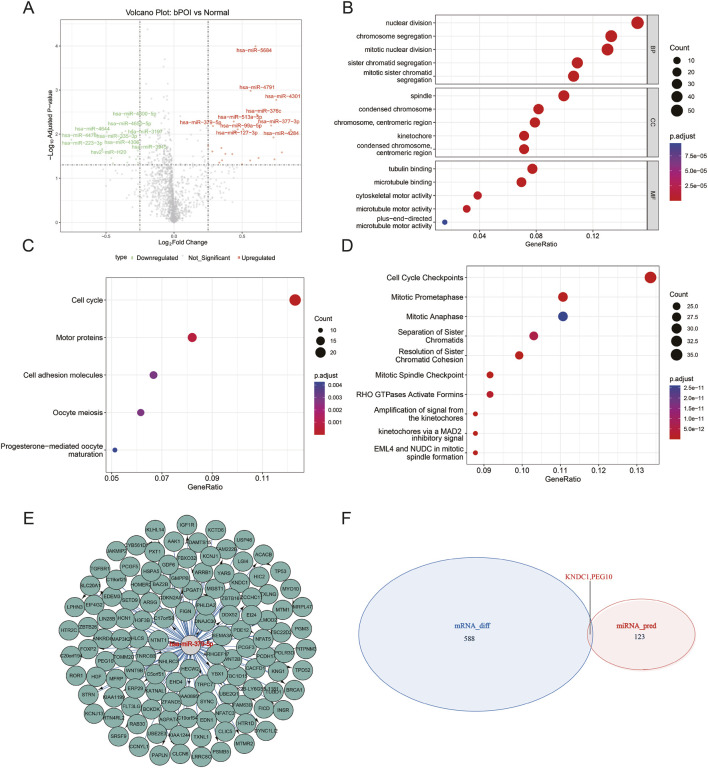
Identification of DEMs and target gene analysis. **(A)** Volcano plot of DEMs; **(B)** GO enrichment analysis of targets of the DEMs; **(C)** KEGG pathway enrichment analysis of targets of the DEMs; **(D)** Reactome pathway enrichment analysis of targets of the DEMs; **(E)** Regulatory network of miR-379-5p and its target mRNAs; **(F)** Venn diagram showing overlap between DEMs and predicted target genes of miR-379-5p.

To systematically investigate the regulatory mechanisms of miRNAs in the pathogenesis of POI, we conducted an in-depth analysis of the miRNA expression profiles associated with POI. By intersection results from edgeR, limma, and DESeq2, we identified a total of 610 DEMs, including 221 downregulated and 389 upregulated miRNAs. The volcano plot for DEMs showed that miRNAs such as miR-5684, miR-4791, miR-4301, and miR-379-5p were significantly upregulated, while miRNAs like miR-4800-5p, miR-4476, and miR-223-3p were significantly downregulated (Figure 2A). To clarify the potential biological functions of the DEMs, we performed a function enrichment analysis of their predicted target genes. GO, KEGG, and Reactome analyses consistently indicated that the target genes of DEMs were enriched in biological processes such as mitotic nuclear division, motor proteins and cell adhesion molecules, as well as cell cycle checkpoints (Figures 2B–D). These results were highly consistent with analysis of the DEMs, collectively suggesting that cell cycle disruption is one of the core pathological mechanisms involved in POI.

Previous studies have demonstrated that miR-379-5p can modulate the proliferative capacity and cell cycle progression of tumor cells and enhance drug resistance (Meng et al., 2023; Shukla et al., 2025; Yan et al., 2022). Therefore, we selected it as a candidate molecule for in-depth analysis. The regulatory network revealed that miR-379-5p could interact with 125 mRNAs (Figure 2E). Further Venn diagram analysis identified two overlapped genes KNDC1 and PEG10 between the DEGs and the predicted target genes of miR-379-5p (Figure 2F). This finding suggests that KNDC1 and PEG10 might serve as key targets of miR-379-5p in POI, providing direction for subsequent experimental validation.

### 3.3 miR-379-5p targets and inhibits KNDC1 and PEG10 expression

**FIGURE 3:**
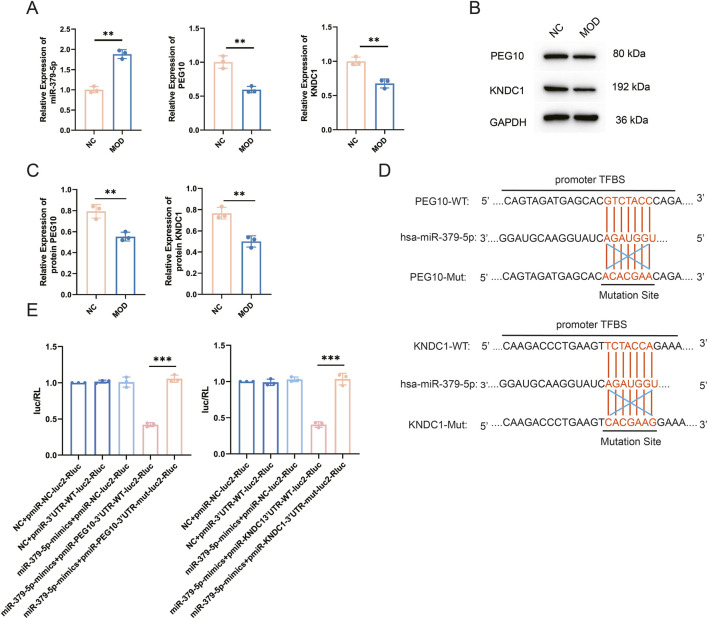
miR-379-5p downregulates KNDC1 and PEG10 expression. **(A)** qRT-PCR was performed to quantify the mRNA levels of miR-379-5p, KNDC1, and PEG10 in the NC and MOD group; **(B)** Protein expression of KNDC1 and PEG10 in the NC group and MOD group was detected by Western blotting; **(C)** Quantitative analysis of band intensities from Western blotting; **(D)** Schematic diagram of the dual-luciferase reporter constructs; **(E)** Statistical results of relative luciferase activity. All data are presented as mean ± standard deviation from three independent experiments (n = 3). Data in A and C were analyzed for statistical significance using an unpaired Student’s t-test; data in E were analyzed using one-way ANOVA followed by Tukey’s multiple comparison test. **P < 0.01, ***P < 0.001.

Several studies have shown that VCD can induce POI models both *in vivo* and *in vitro* (Dou et al., 2022; Li et al., 2025a; Li et al., 2025b). In the present study, the cytotoxic effect of VCD on KGN cells was dose- and time-dependent (Supplementary Figures S1A,B). Treatment of KGN cells with 2 mM VCD for 24 h (designated as the MOD group) resulted in a significant decrease in estradiol (E2) levels in the culture medium (Supplementary Figure S1C). Meanwhile, the expression of estrogen synthesis-related genes, including steroidogenic acute regulatory protein (StAR), cytochrome P450 family 19 subfamily A member 1 (CYP19A1), and follicle-stimulating hormone receptor (FSHR), was markedly downregulated (Supplementary Figure S1D). In addition, compared with the NC group, the MOD group exhibited a significantly higher apoptosis rate and cell cycle arrest at the G0/G1 phase (Supplementary Figures S1E,F). These data collectively indicate the successful establishment of a POI granulosa cell (GC) model. To confirm the bioinformatically predicted high expression of miR-379-5p in POI GCs, we performed experimental verification in the aforementioned POI cell model. qRT-PCR analysis showed that miR-379-5p expression was significantly upregulated in the MOD group compared to the NC group, whereas the mRNA levels of KNDC1 and PEG10 were markedly suppressed (Figure 3A). Western blot analysis further validated the marked reduction in KNDC1 and PEG10 protein expression in the MOD group (Figures 3B,C), consistent with the observed changes at the mRNA level.

To clarify the direct targeting relationship between miR-379-5p and KNDC1/PEG10, we cloned both the wild-type and mutant sequences containing the miR-379-5p binding sites into dual luciferase reporter vectors (Figure 3D). Results showed that transfection with miR-379-5p mimics specifically suppressed luciferase activity in the KNDC1-WT and PEG10-WT reporter vectors, while their mutant counterparts remained largely unaffected (Figure 3E). These results demonstrate that miR-379-5p is upregulated in the MOD group and directly targets and suppresses KNDC1 and PEG10.

### 3.4 miR-379-5p induces apoptosis and cell cycle arrest in GCs

**FIGURE 4:**
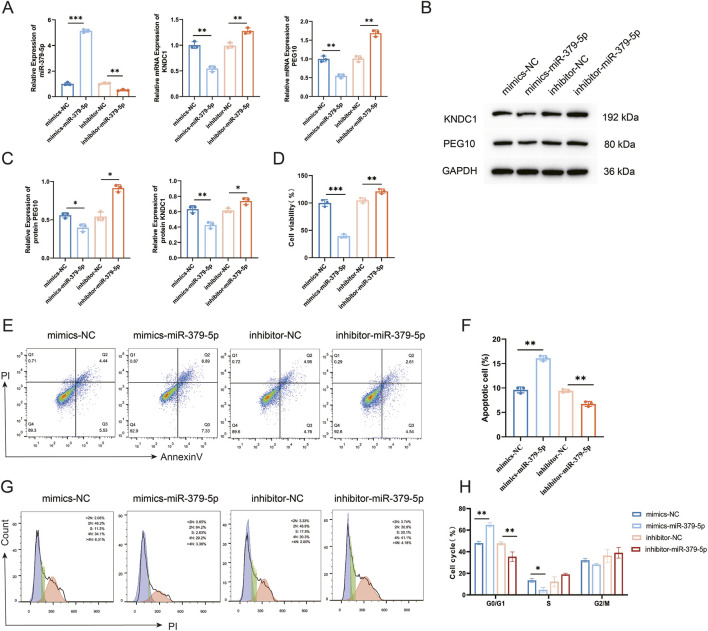
miR-379-5p promotes apoptosis and cell cycle arrest in GCs. **(A)** The mRNA levels of miR-379-5p, KNDC1, and PEG10 in GCs were assessed by qRT-PCR; **(B)** Protein levels of KNDC1 and PEG10 in GCs were examined by Western blotting; **(C)** Quantitative analysis of Western blot band intensities; **(D)** Cell viability of GCs was measured by CCK-8 assay; **(E)** Apoptosis of GCs was detected by flow cytometry; **(F)** Quantification of apoptosis rate; **(G)** Cell cycle profiles of GCs was determined by flow cytometry; **(H)** Quantitative analysis of cell cycle distribution. All data are presented as mean ± standard deviation from three independent experiments (n = 3). Data were analyzed using one-way ANOVA followed by Tukey’s multiple comparison test. *P < 0.05, **P < 0.01, ***P < 0.001.

To elucidate the biological function of miR-379-5p in GCs, we assessed its effects on apoptosis and cell cycle progression by using miR-379-5p mimics or inhibitors. qRT-PCR results confirmed that transfection with miR-379-5p mimics significantly increased its expression, while the inhibitor effectively reduced it, indicating the successful establishment of the intervention model (Figure 4A). Concurrently, the introduction of miR-379-5p mimics markedly downregulated the mRNA and protein expression levels of KNDC1 and PEG10, whereas inhibition of miR-379-5p reversed this effect (Figures 4A–C), further validating its role as a negative regulator of these target genes.

In terms of cellular function, miR-379-5p mimics significantly inhibited cell viability, while its inhibition promoted recovery of cell viability, as confirmed by the CCK-8 assay (Figure 4D). Flow cytometry analysis revealed that transfection with miR-379-5p mimics markedly elevated the apoptotic rate, which was reduced following its inhibition (Figures 4E,F). Furthermore, cell cycle analysis indicated that miR-379-5p mimics induced G0/G1 phase arrest, as evidenced by G0/G1 accumulation coupled with S phase reduction. Inhibition of miR-379-5p reversed this phenomenon (Figures 4G,H). In summary, miR-379-5p not only negatively regulated KNDC1 and PEG10 expression but also suppressed cell proliferation, induced apoptosis, and caused G0/G1 phase arrest, suggesting its potential role in ovarian function decline.

### 3.5 miR-379-5p promotes apoptosis in OGCs by suppressing KNDC1 and PEG10 expression

**FIGURE 5:**
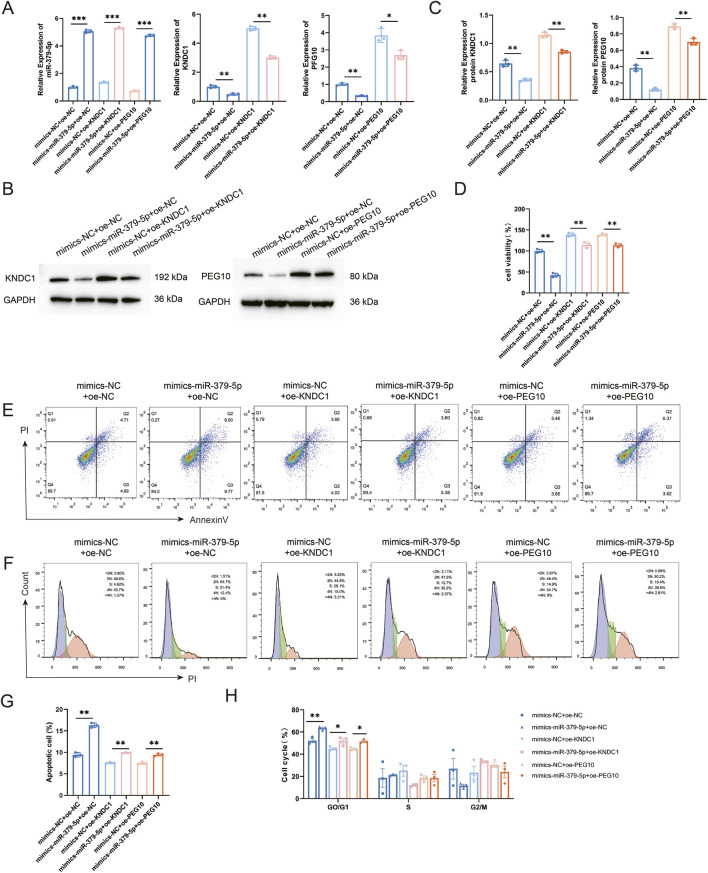
miR-379-5p Induces apoptosis and cell cycle arrest in GCs by suppressing KNDC1 and PEG10 expression. **(A)** The mRNA levels of miR-379-5p, KNDC1, and PEG10 in GCs were assessed by qRT-PCR; **(B)** Protein levels of KNDC1 and PEG10 in GCs were examined by Western blotting; **(C)** Quantitative analysis of Western blotting band intensities; **(D)** Cell viability of GCs was measured by CCK-8 assay; **(E)** Apoptosis of GCs was analyzed by flow cytometry; **(F)** Cell cycle distribution was analyzed by flow cytometry; **(G)** Quantitative analysis of apoptosis rate; **(H)** Quantitative analysis of cell cycle distribution. All data are presented as mean ± standard deviation from three independent experiments (n = 3). Data were analyzed using one-way ANOVA followed by Tukey’s multiple comparison test. *P < 0.05, **P < 0.01, ***P < 0.001.

To determine whether miR-379-5p mediates its pro-apoptotic and cell cycle arrest effects by targeting KNDC1 and PEG10, we performed rescue experiments. Following transfection with miR-379-5p mimics, KNDC1 or PEG10 overexpression plasmids were introduced separately. qRT-PCR and Western blotting confirmed that the downregulation of target genes induced by miR-379-5p, at both mRNA and protein levels, was effectively reversed by overexpressing either gene (Figures 5A–C). CCK-8 assay indicated that overexpression of KNDC1 or PEG10 significantly alleviated the suppression of cell viability caused by miR-379-5p (Figure 5D). Flow cytometry further demonstrated that increased apoptosis rate resulting from miR-379-5p mimics was reversed upon overexpression of either KNDC1 or PEG10 (Figures 5E,G). Meanwhile, cell cycle analysis revealed that co-transfection with KNDC1 or PEG10 also markedly mitigated the G0/G1 phase arrest induced by miR-379-5p (Figures 5F,H). These results collectively confirm that miR-379-5p induces apoptosis and cell cycle arrest in OGCs by repressing its target genes, KNDC1 and PEG10.

## 4 Discussion

In this study, we integrated mRNA and miRNA microarray datasets related to POI from the GEO database, constructed a miRNA-mRNA regulatory network, and elucidated the mechanism by which miR-379-5p induced cell apoptosis and cycle arrest in GCs by targeting KNDC1 and PEG10.

Depletion of the primordial follicle pool, accelerated oocyte loss, and GC dysfunction represent fundamental pathological features of POI (Imbar and Eisenberg, 2014; Edson et al., 2009). As key post-transcriptional regulators, miRNAs play important roles in maintaining ovarian homeostasis (Zhang et al., 2023). For example, FOXL2-targeting miR-133b regulates steroidogenesis (Liu et al., 2019), while miR-146b-5p drives GCs senescence and POI progression via dual mechanisms: blockade of the Dab2ip/Ask1/p38-Mapk cascade and interference with H2AX phosphorylation (Liu et al., 2021). However, there is still a lack of research focused on constructing POI-specific miRNA-mRNA regulatory networks from a systematic perspective, particularly in screening key hub molecules within these networks. Based on combined analysis of POI-related mRNA and miRNA expression profiles, this study found that DEGs were predominantly mapped to biological processes, including cell cycle regulation and chromosome segregation. This finding suggests that cell cycle disruption is a core pathological feature of POI. Consequently, we prioritized the notably upregulated miR-379-5p in POI for further investigation.

In recent years, the role of miR-379-5p in ovarian function regulation has been gradually elucidated. Studies have shown that miR-379-5p is significantly upregulated in GCs from POI patients, where it suppresses DNA repair and cell proliferation by targeting PARP1 and XRCC6 (Dang et al., 2018). Moreover, its regulatory function exhibits notable characteristics that depend on follicular stage. For instance, in a PCOS model, androgens induce the release of exosomal miR-379-5p from preantral follicle GCs, reducing intracellular miR-379-5p levels and relieving PDK1 inhibition, thereby promoting cell proliferation (Salehi et al., 2023). Conversely, at the antral follicle stage, miR-379-5p targets PDK1 to regulate macrophage polarization, which in turn inhibits GCs proliferation (Sale et al., 2023). These findings support the notion that miR-379-5p is an important regulator of ovarian physiological and pathological processes, but its specific targets and regulatory network in the context of POI remain to be explored. Previous studies have implicated miR-379-5p in modulating cell cycle checkpoints and apoptosis in other diseases including tumors (Shukla et al., 2025; Zhang et al., 2022). Nonetheless, its specific targets and functional mechanisms in POI GCs remain largely unexplored. Through intersection analysis, this study identified KNDC1 and PEG10 as direct targets of miR-379-5p in the POI context for the first time and this identification was further confirmed through dual-luciferase reporter assays.

KNDC1, a scaffold protein, is essential for neuronal development and cell proliferation (Kornelius et al., 2011; Mees et al., 2005). Recent studies have found that KNDC1 mediates the Ras/MAPK signaling pathway across various tissues, thereby influencing cell proliferation and apoptosis (Chang et al., 2014). In this study, KNDC1 was downregulated in OGCs, and overexpression of KNDC1 partially reversed miR-379-5p-induced apoptosis and cycle arrest, suggesting that KNDC1 has a protective role in maintaining OGCs homeostasis. Similarly, PEG10, a gene derived from retrotransposons, is known for its high expression in placental development and various cancers, where it is involved in cell cycle and apoptosis regulation (Ribarska et al., 2012). This study provided initial evidence of decreased expression of PEG10 in OGCs, with overexpression of PEG10 alleviating miR-379-5p-induced G0/G1 phase arrest, further supporting its role in promoting cell cycle progression. Functional cell experiments confirmed that miR-379-5p mimics promoted apoptosis, suppressed proliferative capacity, and induced G0/G1 phase arrest in POI GCs by downregulating the expression of KNDC1 and PEG10. These results corroborate earlier studies demonstrating that both KNDC1 and PEG10 play significant roles in regulating cell survival and cell cycle progression in various diseases (Kornelius et al., 2011; Huang et al., 2011; Akamatsu et al., 2015).

Notably, GC dysfunction is reflected not only in decreased cell viability but also in impaired steroid hormone synthesis capacity (Gao et al., 2025). Multiple studies have shown that miRNAs are involved in regulating this process. For example, miR-132 inhibits progesterone production by suppressing StAR while simultaneously promoting the expression of steroidogenic enzymes 3β-hydroxysteroid dehydrogenase (HSD) and 20α-hydroxysteroid dehydrogenase (20α-HSD) (Pankiewicz et al., 2021); miR-320a inhibits steroidogenesis in cumulus cells by targeting CYP11A1 and CYP19A1 (Zhang et al., 2017). Given the marked inhibitory effect of miR-379-5p on GCs proliferation and its capacity to induce cell cycle arrest, it is worthwhile to investigate if it also affects hormone secretion by interfering with steroidogenic enzymes or upstream signaling pathways (such as FSHR-mediated signaling), as a clinically relevant research direction. However, this study did not directly measure hormone secretion, which constitutes one of its limitations.

In addition, this study lacked primary GCs from POI patients and the use of VCD-induced KGN cells as a POI GCs model may introduce some bias in terms of cell proliferation characteristics and metabolic profiles. Furthermore, this study mainly relied on bioinformatics screening and *in vitro* cellular experiments, lacking *in vivo* validation in animal models. Future studies should focus on collecting primary GCs from follicular fluid of POI patients for clinical validation and using animal models to further elucidate the specific roles of the miR-379-5p/KNDC1/PEG10 axis in follicular development and ovarian reserve depletion. At the same time, the specific downstream signaling cascades of KNDC1 and PEG10 need to be further clarified.

## 5 Conclusion

This study characterized the altered expression patterns of DEGs and DGMs in POI, revealing that miR-379-5p promotes apoptosis and cycle arrest in OGCs through dual targeting KNDC1 and PEG10. This research enhances our comprehension of the molecular mechanisms behind POI and provides a new theoretical basis for developing diagnostic and therapeutic strategies targeting miR-379-5p.

## Data Availability

The datasets utilized in this study are publicly available in the GEO repository with the accession codes GSE201276 and GSE100238.
