# Supplementary material for: miR-379-5p promotes ovarian granulosa cell apoptosis in primary ovarian insufficiency by targeting KNDC1 and PEG10
Source: Front Genet. 2026 May 13;17:1827032. doi: 10.3389/fgene.2026.1827032 (PMC13211853; doi:10.3389/fgene.2026.1827032)
Supplement: Supplementary file 1 [file DataSheet1.zip › Supplementary Materials/supplementary materials S2.pdf]

hsa-miR-379-5p：
UGGUAGACUAUGGAACGUAGG

binding site：TCTACCA
mutant site：CACGAAG

KNDC1-WT
GCTAGCGACTGGCCAGGTCCAAAGGGGTCGGCCCAGCCTTGTCCCCCGGCCCAGC
CGGATTCCAGAGCTGCAGCCCCGGCTGGTGCAGCGCCTTCTACGAGGCCGACTGC
TTCGGGGCCGACGTCCACAACTACGTGAAGGACCTGGGGCGGCAGCAGGCGGAC
GGGGCCCTGCCCGACGCCCAGAGCCCGGAGCTGGAACAGCAGCTCATGATGGAG
AAAAGAAACTACCGCAAGACCCTGAAGTTCTACCAGAAACTCTTACAGAAGGAA
AAGAGGAACAAAGGTTCCGACGTCAAGACCATGCTGTCCAAGCTGAAAGGGCAG
CTAGAAGAAATGAAATCCAGGGTGCAATTCCTCAGCTTGGTCAAGAAGTATCTGC
AGGTCATGTACGCGGAACGCTGGGGCCTGGAGCCCTGCACCCTCCCAGTGATCGT
GAACATCGCGGCCGCACCCTGCGACACGCTGGACTTCAGCCCCCTGGACGAGTCC
TCCCTCGAG

KNDC1-Mut
GCTAGCGACTGGCCAGGTCCAAAGGGGTCGGCCCAGCCTTGTCCCCCGGCCCAGC
CGGATTCCAGAGCTGCAGCCCCGGCTGGTGCAGCGCCTTCTACGAGGCCGACTGC
TTCGGGGCCGACGTCCACAACTACGTGAAGGACCTGGGGCGGCAGCAGGCGGAC
GGGGCCCTGCCCGACGCCCAGAGCCCGGAGCTGGAACAGCAGCTCATGATGGAG
AAAAGAAACTACCGCAAGACCCTGAAGTCACGAAGGAAACTCTTACAGAAGGAA
AAGAGGAACAAAGGTTCCGACGTCAAGACCATGCTGTCCAAGCTGAAAGGGCAG
CTAGAAGAAATGAAATCCAGGGTGCAATTCCTCAGCTTGGTCAAGAAGTATCTGC
AGGTCATGTACGCGGAACGCTGGGGCCTGGAGCCCTGCACCCTCCCAGTGATCGT
GAACATCGCGGCCGCACCCTGCGACACGCTGGACTTCAGCCCCCTGGACGAGTCC
TCCCTCGAG

binding site： GTCTACC
mutant site： ACACGAA

PEG10-WT
GCTAGCGTCGTCCTAGGGGTTCGCTGGCTGAGCACACATGATCCCAATATCACAT
GGAGCACTCGATCTATCGTCTTTGATTCTGAATACTGCCGCTACCACTGCCGGAT
GTATTCTCCAATACCACCATCGCTCCCACCACCAGCACCACAACCGCCACTCTAT
TATCCAGTAGATGGATACAGAGTTTACCAACCAGTGAGGTATTACTATGTCCAGA
ATGTGTACACTCCAGTAGATGAGCACGTCTACCCAGATCACCGCCTGGTTGACCC
TCACATAGAAATGATACCTGGAGCACACAGTATTCCCAGTGGACATGTGTATTCA
CTGTCCGAACCTGAAATGGCAGCTCTTCGAGATTTTGTGGCAAGAAATGTAAAAG
ATGGGCTAATTACTCCAACGATTGCACCTAATGGAGCCCAAGTTCTCCAGGTGAA
GAGGGGGTGGAAACTGCAAGTTTCTTATGATTGCCGAGCTCCAAACAATTTTACC
TCGAG

PEG10-Mut
GCTAGCGTCGTCCTAGGGGTTCGCTGGCTGAGCACACATGATCCCAATATCACAT
GGAGCACTCGATCTATCGTCTTTGATTCTGAATACTGCCGCTACCACTGCCGGAT
GTATTCTCCAATACCACCATCGCTCCCACCACCAGCACCACAACCGCCACTCTAT
TATCCAGTAGATGGATACAGAGTTTACCAACCAGTGAGGTATTACTATGTCCAGA
ATGTGTACACTCCAGTAGATGAGCACACACGAACAGATCACCGCCTGGTTGACCC
TCACATAGAAATGATACCTGGAGCACACAGTATTCCCAGTGGACATGTGTATTCA
CTGTCCGAACCTGAAATGGCAGCTCTTCGAGATTTTGTGGCAAGAAATGTAAAAG
ATGGGCTAATTACTCCAACGATTGCACCTAATGGAGCCCAAGTTCTCCAGGTGAA
GAGGGGGTGGAAACTGCAAGTTTCTTATGATTGCCGAGCTCCAAACAATTTTACC
TCGAG
