# Supplementary figures and images for: miR-379-5p promotes ovarian granulosa cell apoptosis in primary ovarian insufficiency by targeting KNDC1 and PEG10
Source: Front Genet. 2026 May 13;17:1827032. doi: 10.3389/fgene.2026.1827032 (PMC13211853; doi:10.3389/fgene.2026.1827032)

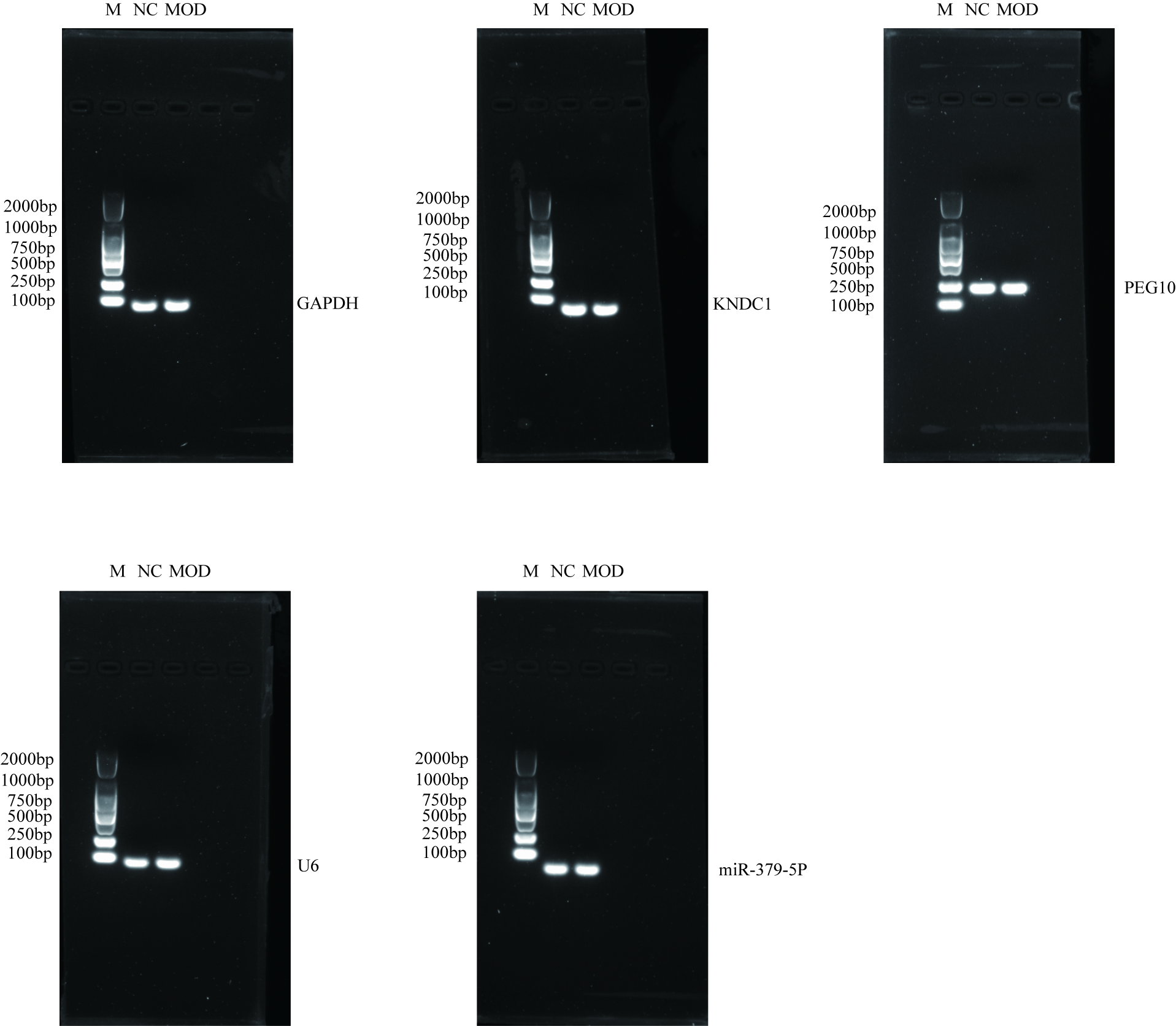

Supplement: Supplementary file 1 [file DataSheet1.zip › Supplementary Materials/supplementary materials S1/Agarose Gel Electrophoresis Image.tif]

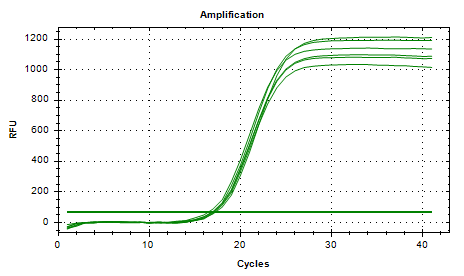

Supplement: Supplementary file 1 [file DataSheet1.zip › Supplementary Materials/supplementary materials S1/Figure 3A ¿C Amplification curves and melting curves/GAPDH-Amplification curves.png]

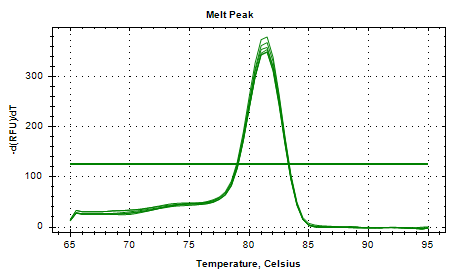

Supplement: Supplementary file 1 [file DataSheet1.zip › Supplementary Materials/supplementary materials S1/Figure 3A ¿C Amplification curves and melting curves/GAPDH-Melting curves.png]

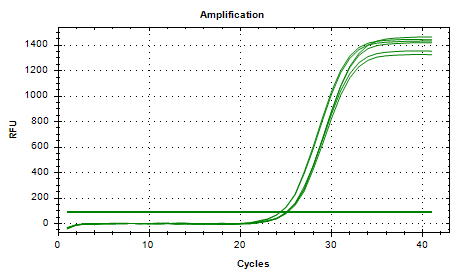

Supplement: Supplementary file 1 [file DataSheet1.zip › Supplementary Materials/supplementary materials S1/Figure 3A ¿C Amplification curves and melting curves/KNDC1-Amplification curves.png]

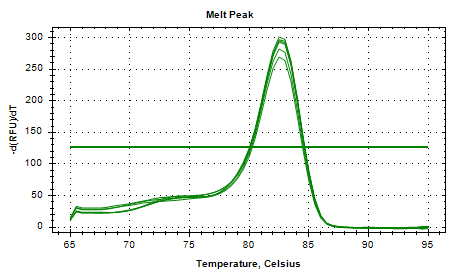

Supplement: Supplementary file 1 [file DataSheet1.zip › Supplementary Materials/supplementary materials S1/Figure 3A ¿C Amplification curves and melting curves/KNDC1-Melting curves.png]

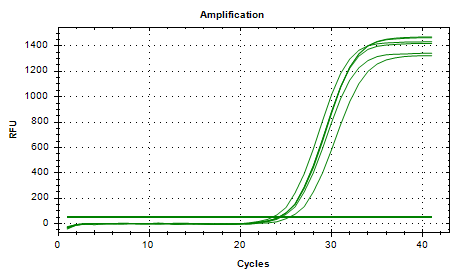

Supplement: Supplementary file 1 [file DataSheet1.zip › Supplementary Materials/supplementary materials S1/Figure 3A ¿C Amplification curves and melting curves/miR-379-5p-Amplification curves.png]

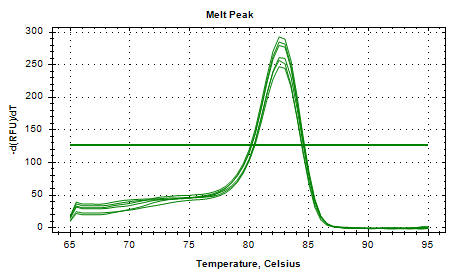

Supplement: Supplementary file 1 [file DataSheet1.zip › Supplementary Materials/supplementary materials S1/Figure 3A ¿C Amplification curves and melting curves/miR-379-5p-Melting curves.png]

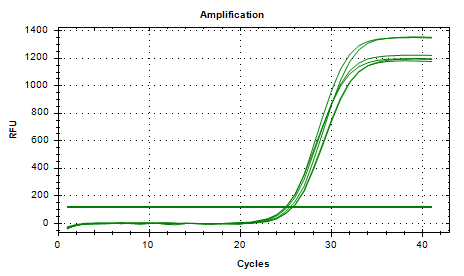

Supplement: Supplementary file 1 [file DataSheet1.zip › Supplementary Materials/supplementary materials S1/Figure 3A ¿C Amplification curves and melting curves/PEG10-Amplification curves.png]

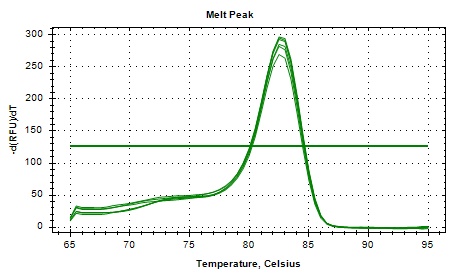

Supplement: Supplementary file 1 [file DataSheet1.zip › Supplementary Materials/supplementary materials S1/Figure 3A ¿C Amplification curves and melting curves/PEG10-Melting curves.png]

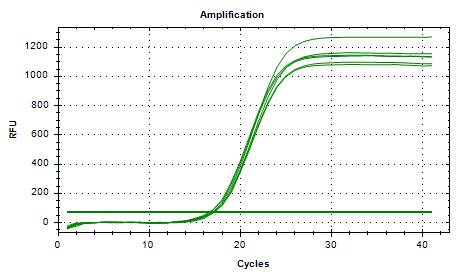

Supplement: Supplementary file 1 [file DataSheet1.zip › Supplementary Materials/supplementary materials S1/Figure 3A ¿C Amplification curves and melting curves/U6-Amplification curves.png]

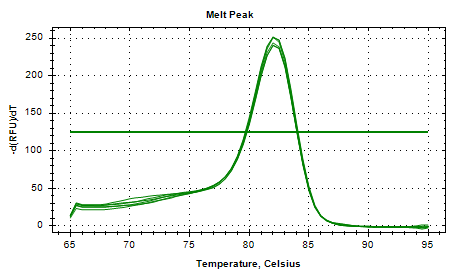

Supplement: Supplementary file 1 [file DataSheet1.zip › Supplementary Materials/supplementary materials S1/Figure 3A ¿C Amplification curves and melting curves/U6-Melting curves.png]

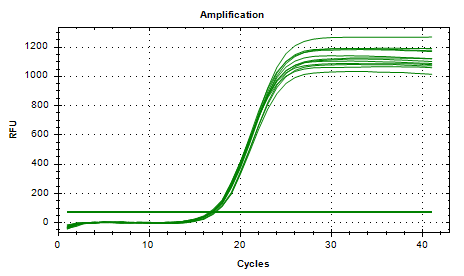

Supplement: Supplementary file 1 [file DataSheet1.zip › Supplementary Materials/supplementary materials S1/Figure 4A ¿C Amplification curves and melting curves/GAPDH-Amplification curves.png]

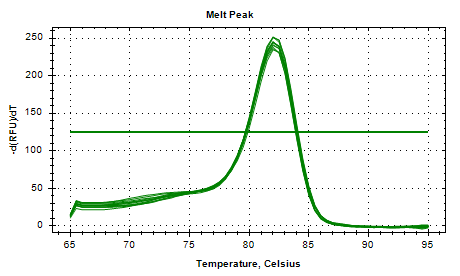

Supplement: Supplementary file 1 [file DataSheet1.zip › Supplementary Materials/supplementary materials S1/Figure 4A ¿C Amplification curves and melting curves/GAPDH-Melting curves.png]

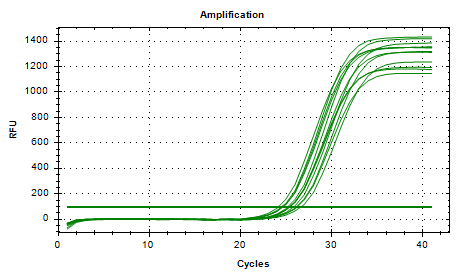

Supplement: Supplementary file 1 [file DataSheet1.zip › Supplementary Materials/supplementary materials S1/Figure 4A ¿C Amplification curves and melting curves/KNDC1-Amplification curves.png]

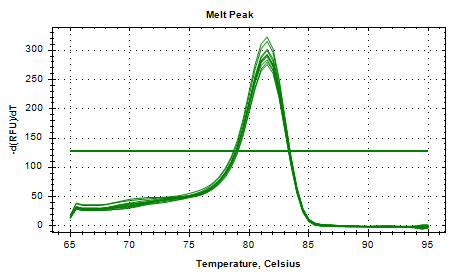

Supplement: Supplementary file 1 [file DataSheet1.zip › Supplementary Materials/supplementary materials S1/Figure 4A ¿C Amplification curves and melting curves/KNDC1-Melting curves.png]

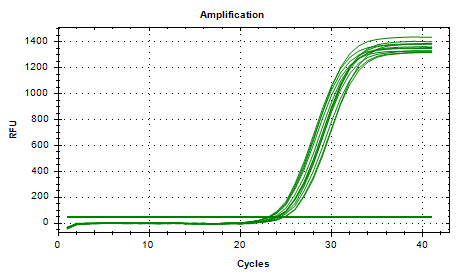

Supplement: Supplementary file 1 [file DataSheet1.zip › Supplementary Materials/supplementary materials S1/Figure 4A ¿C Amplification curves and melting curves/miR-379-5p-Amplification curves.png]

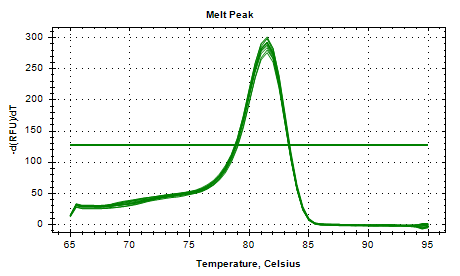

Supplement: Supplementary file 1 [file DataSheet1.zip › Supplementary Materials/supplementary materials S1/Figure 4A ¿C Amplification curves and melting curves/miR-379-5p-Melting curves.png]

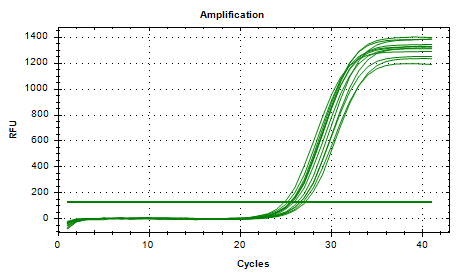

Supplement: Supplementary file 1 [file DataSheet1.zip › Supplementary Materials/supplementary materials S1/Figure 4A ¿C Amplification curves and melting curves/PEG10-Amplification curves.png]

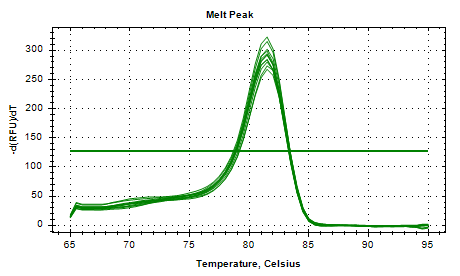

Supplement: Supplementary file 1 [file DataSheet1.zip › Supplementary Materials/supplementary materials S1/Figure 4A ¿C Amplification curves and melting curves/PEG10-Melting curves.png]

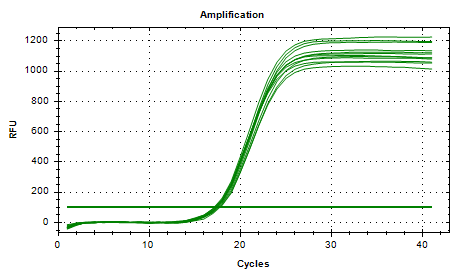

Supplement: Supplementary file 1 [file DataSheet1.zip › Supplementary Materials/supplementary materials S1/Figure 4A ¿C Amplification curves and melting curves/U6-Amplification curves.png]

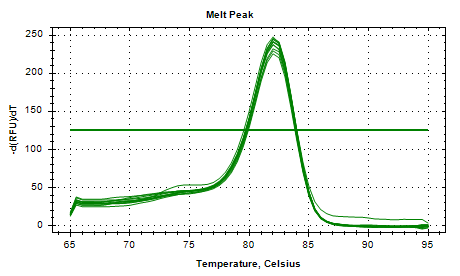

Supplement: Supplementary file 1 [file DataSheet1.zip › Supplementary Materials/supplementary materials S1/Figure 4A ¿C Amplification curves and melting curves/U6-Melting curves.png]

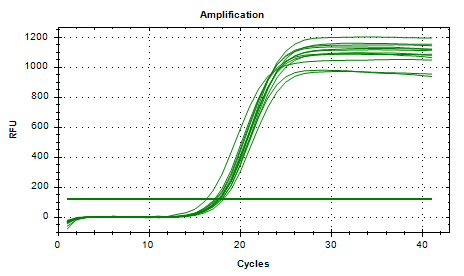

Supplement: Supplementary file 1 [file DataSheet1.zip › Supplementary Materials/supplementary materials S1/Figure 5A ¿C Amplification curves and melting curves/GAPDH-Amplification curves.png]

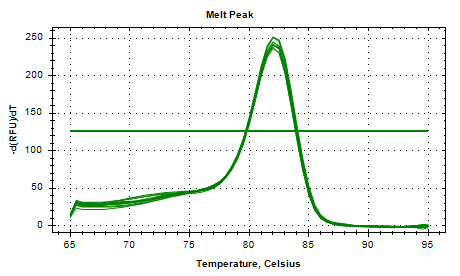

Supplement: Supplementary file 1 [file DataSheet1.zip › Supplementary Materials/supplementary materials S1/Figure 5A ¿C Amplification curves and melting curves/GAPDH-Melting curves.png]

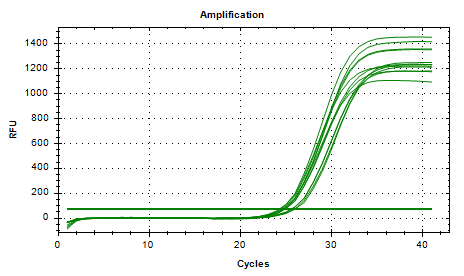

Supplement: Supplementary file 1 [file DataSheet1.zip › Supplementary Materials/supplementary materials S1/Figure 5A ¿C Amplification curves and melting curves/KNDC1-Amplification curves.png]

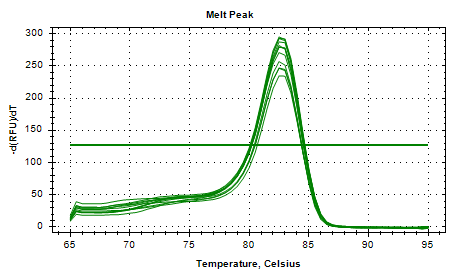

Supplement: Supplementary file 1 [file DataSheet1.zip › Supplementary Materials/supplementary materials S1/Figure 5A ¿C Amplification curves and melting curves/KNDC1-Melting curves.png]

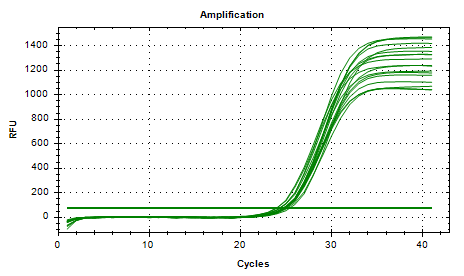

Supplement: Supplementary file 1 [file DataSheet1.zip › Supplementary Materials/supplementary materials S1/Figure 5A ¿C Amplification curves and melting curves/miR-379-5p-Amplification curves.png]

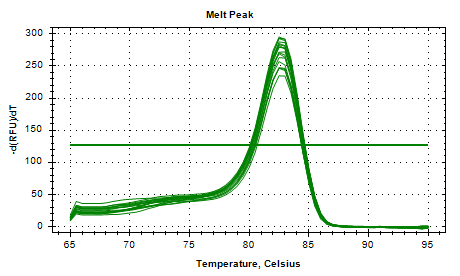

Supplement: Supplementary file 1 [file DataSheet1.zip › Supplementary Materials/supplementary materials S1/Figure 5A ¿C Amplification curves and melting curves/miR-379-5p-Melting curves.png]

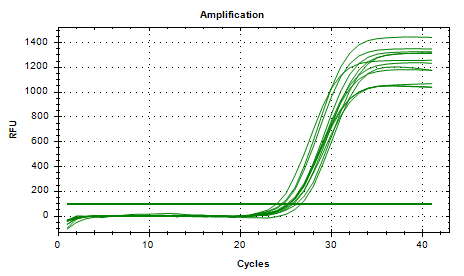

Supplement: Supplementary file 1 [file DataSheet1.zip › Supplementary Materials/supplementary materials S1/Figure 5A ¿C Amplification curves and melting curves/PEG10-Amplification curves.png]

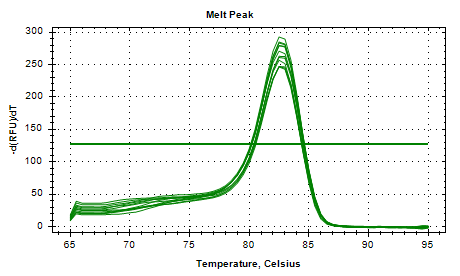

Supplement: Supplementary file 1 [file DataSheet1.zip › Supplementary Materials/supplementary materials S1/Figure 5A ¿C Amplification curves and melting curves/PEG10-Melting curves.png]

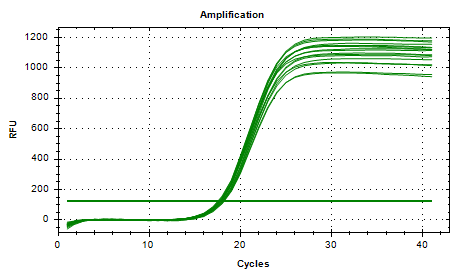

Supplement: Supplementary file 1 [file DataSheet1.zip › Supplementary Materials/supplementary materials S1/Figure 5A ¿C Amplification curves and melting curves/U6-Amplification curves.png]

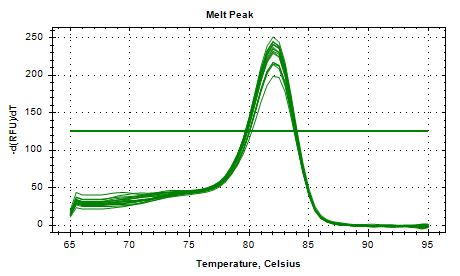

Supplement: Supplementary file 1 [file DataSheet1.zip › Supplementary Materials/supplementary materials S1/Figure 5A ¿C Amplification curves and melting curves/U6-Melting curves.png]

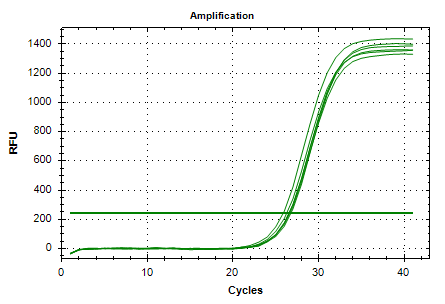

Supplement: Supplementary file 1 [file DataSheet1.zip › Supplementary Materials/supplementary materials S1/Figure S1D ¿C Amplification curves and melting curves/CYP19A1-Amplification curves.png]

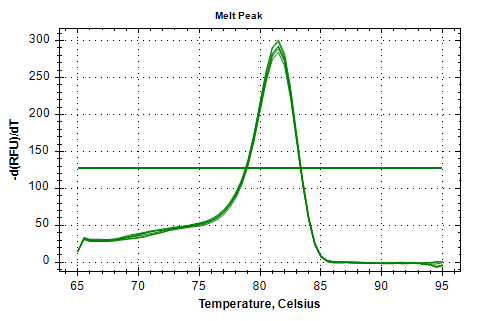

Supplement: Supplementary file 1 [file DataSheet1.zip › Supplementary Materials/supplementary materials S1/Figure S1D ¿C Amplification curves and melting curves/CYP19A1-Melting curves.png]

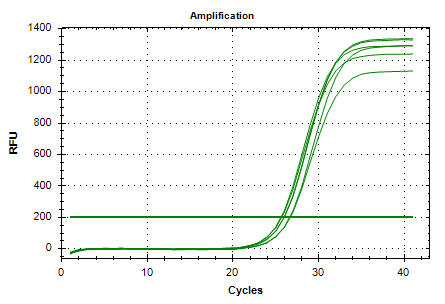

Supplement: Supplementary file 1 [file DataSheet1.zip › Supplementary Materials/supplementary materials S1/Figure S1D ¿C Amplification curves and melting curves/FSHR-Amplification curves.png]

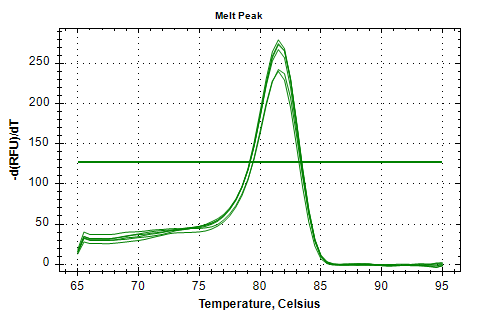

Supplement: Supplementary file 1 [file DataSheet1.zip › Supplementary Materials/supplementary materials S1/Figure S1D ¿C Amplification curves and melting curves/FSHR-Melting curves.png]

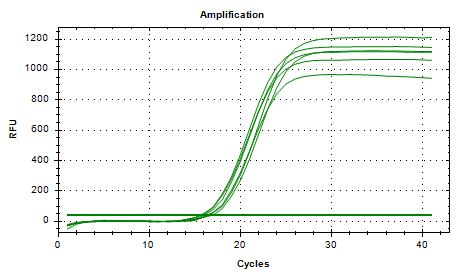

Supplement: Supplementary file 1 [file DataSheet1.zip › Supplementary Materials/supplementary materials S1/Figure S1D ¿C Amplification curves and melting curves/GAPDH-Amplification curves.png]

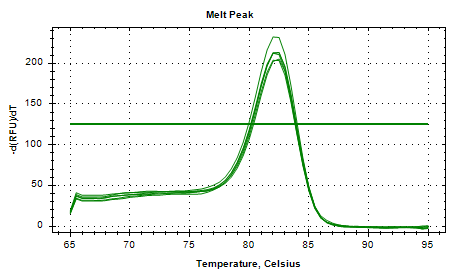

Supplement: Supplementary file 1 [file DataSheet1.zip › Supplementary Materials/supplementary materials S1/Figure S1D ¿C Amplification curves and melting curves/GAPDH-Melting curves.png]

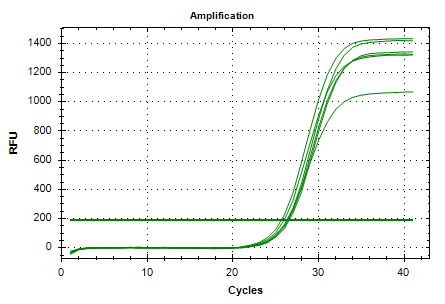

Supplement: Supplementary file 1 [file DataSheet1.zip › Supplementary Materials/supplementary materials S1/Figure S1D ¿C Amplification curves and melting curves/StAR-Amplification curves.png]

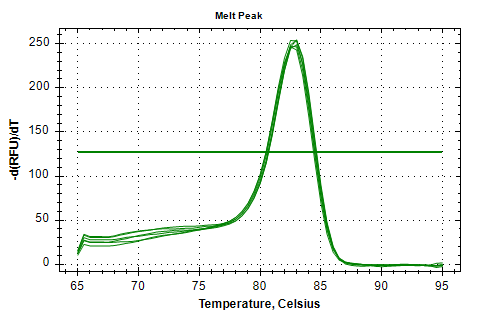

Supplement: Supplementary file 1 [file DataSheet1.zip › Supplementary Materials/supplementary materials S1/Figure S1D ¿C Amplification curves and melting curves/StAR-Melting curves.png]
